# Neurophysiologic and Cognitive Changes Arising from Cognitive Training Interventions in Persons with Mild Cognitive Impairment: A Systematic Review

**DOI:** 10.1155/2018/7301530

**Published:** 2018-12-02

**Authors:** Eliane C. Miotto, Alana X. Batista, Sharon S. Simon, Benjamin M. Hampstead

**Affiliations:** ^1^Department of Neurology, University of Sao Paulo, Brazil; ^2^Cognitive Neuroscience Division, Department of Neurology, Columbia University, USA; ^3^Old Age Research Group, Institute and Department of Psychiatry, University of Sao Paulo, Sao Paulo, Brazil; ^4^Mental Health Service, VA Ann Arbor Healthcare System, Ann Arbor, MI, USA; ^5^Neuropsychology Section, Department of Psychiatry, University of Michigan, USA

## Abstract

**Background:**

Previous reviews have generally reported cognitive//behavioral improvements after cognitively oriented treatments (COTs) in persons with MCI. However, comparatively little is known about the neural mechanisms associated with such cognitive improvement.

**Objective:**

The primary aim of the current review was to examine neurophysiological changes measured by functional magnetic resonance imaging (fMRI) and possible cognitive changes following COTs in those with MCI*. Methods*. An extensive literature search was conducted up to August 2018. Inclusion criteria were (1) studies that evaluated the effects of the COTs in patients with amnestic single- or multiple-domain MCI using fMRI, (2) the MCI patient sample having met Petersen's or Jack/Bond's criteria, (3) randomized and/or controlled trials, (4) fMRI and cognitive assessments completed pre- and post-intervention, and (5) articles available in English.

**Results:**

Amongst the 26 articles found, 7 studies were included according to the above inclusion criteria. A total of 3 studies applied rehearsal-based strategies as the primary intervention, all of which used computerized cognitive training. Four studies used fMRI to investigate the neurophysiologic and cognitive changes associated with memory strategy training. The majority of the studies included in this review showed evidence of improved objective cognitive performance associated with COTs, even in tasks similar to everyday life activities. In addition, there were significant changes in brain activation associated with interventions, in both typical and atypical brain areas and networks related to memory.

**Conclusions:**

Although additional studies are needed given the small sample size, these initial findings suggest that cognitive improvement after COTs is generally associated with both compensatory (i.e., engaging alternative brain regions or networks not “typically” engaged) and restorative (i.e., reengaging the “typical” brain regions or networks) mechanisms.

## 1. Introduction

The recent growth of interest in nonpharmacologic cognitively oriented treatments (COTs), particularly cognitive training, in those with mild cognitive impairment (MCI) has been fueled by a combination of data showing limited benefits of existing pharmacologic agents [1, 2] and recognition that cognitive and lifestyle factors may be protective against disease-related decline [3]. While several reviews have examined the efficacy of COTs in those with MCI and generally found the results to be positive [4–8], less is known about the neural mechanisms associated with such cognitive improvement. Thus, the primary aim of the current review was to examine the neurophysiological and cognitive changes, as measured using functional magnetic resonance imaging (fMRI), following COTs in those with MCI.

A critical first step in understanding such neurophysiological changes is to recognize the heterogeneity that exists in both treatment and fMRI methods. In our previous methodological COT review [9], we classified commonly used approaches based on the presumed cognitive “mechanism of action.” Specifically, we identified a group of approaches that relied primarily on the rehearsal of information (subtracting cues, spaced retrieval, and computerized cognitive training), those that relied on the use of external compensatory methods (e.g., notebooks, calendars), and those that relied on internal compensatory methods (i.e., mnemonic strategies). Our previous research supports such distinctions as, for example, we revealed that mnemonic strategy training (MST) enhanced memory for object location associations significantly more than a tightly matched repeated exposure active control group both 2 days after training and at a 1-month follow-up [10]. MST requires the user to actively hold and manipulate to-be-learned information, processes that engage cognitive control mechanisms such as working memory. As such, we would expect to see increased neurophysiologic functioning in lateral frontoparietal regions that mediate such cognitive control processes. In contrast, fMRI studies of repeated exposure have been shown to result in a repetition suppression effect [11], which is characterized as reduced blood oxygen level-dependent (BOLD) signal and typically interpreted as evidence of enhanced processing efficiency. Thus, rehearsal-based COTs may result in fundamentally different patterns of neurophysiologic change relative to MST. Therefore, we maintain that the type of COT is critical to consider when evaluating outcomes (especially neurophysiological).

The second important source of heterogeneity is the type of fMRI used to evaluate COT effects. While task-based fMRI paradigms were the standard for the first decades of fMRI research, recent years have seen a shift toward methods that use “resting-state” fMRI (rs-fMRI) to evaluate within- and between-network connectivity. While task-based paradigms identify patterns of BOLD signal directly arising from task performance, rs-fMRI relies on inherent low-frequency oscillations and is dependent on correlations with cognitive/behavioral performance. However, task-based paradigms differ markedly across studies and may yield different patterns of activation due to nothing more than the nature of the stimuli used (e.g., verbal vs. visuospatial). In contrast, rs-fMRI can be easily implemented and standardized across sessions and locations but, again, analytic methods vary widely. These relative strengths and weaknesses are critical to consider when examining the effects of COTs and reinforce the need for a more nuanced review of the available literature.

With the above noted caveats in mind, the current review specifically addressed the following questions: (1) Which types of COT have been applied to MCI patients? (2) Can COT effects in persons with MCI be generalized/transferred to objective cognitive measurements? (3) Do the observed changes represent evidence of compensation (i.e., engaging alternative brain regions or networks not “typically” engaged) and/or restoration (i.e., reengaging the “typical” brain regions or networks)?

## 2. Methods

### 2.1. Review Strategy

Studies focusing on the main objective of this systematic review published up to August 2018 were included. We selected only those studies that fulfilled the following criteria: (1) studies that primarily evaluated the effects of the cognitive training in patients with amnestic single- or multiple-domain MCI using fMRI, (2) the MCI patient sample having met Petersen or Albert's criteria (single cognitive test impaired per domain, >1.5 SD below expectations) [12] or Jack/Bondi's criteria (two tests impaired per domain, >1 SD below norms) [13], (3) controlled trials and case series, (4) fMRI and cognitive assessments completed pre- and post-intervention, and (5) articles available in English.

Databases included were PubMed, Medline, and Google Scholar. The search terms were specified to be found in the title of the studies and were (1) mild cognitive impairment, (2) cognitive training, (3) attention training, (4) executive training, (5) memory training, (6) and fMRI. The search terms combinations in the database were (1) + (6) + (2), (1) + (6) + (3), (1) + (6) + (4), and (1) + (6) + (5).

**Figure 1:**
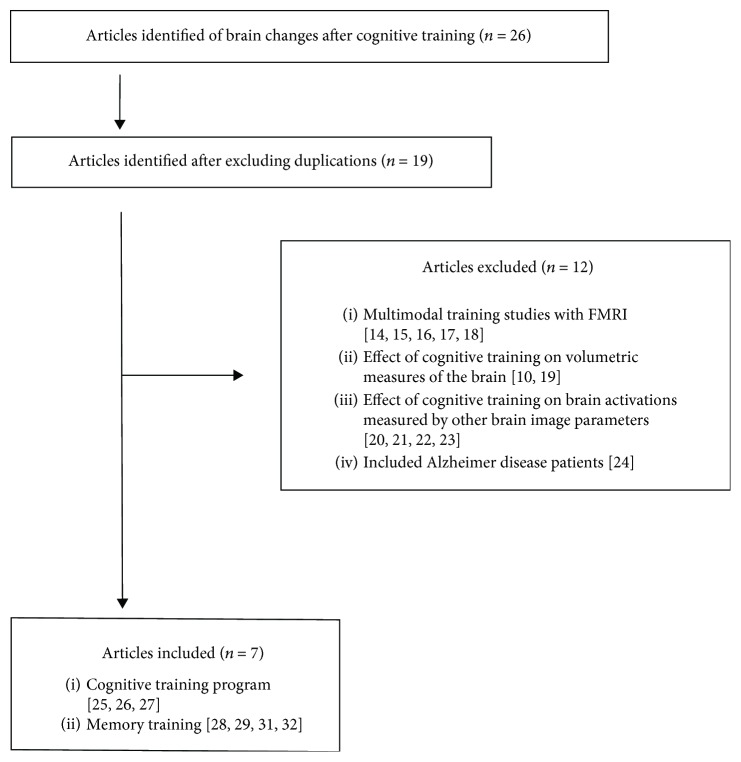
Summary of the studies identified and included in the review.

A summary of the study selection is shown in Figure 1, and the results of the studies included in the current review are shown in Table 1. From the 26 articles found, 10 were excluded due to duplication. Amongst the remained 19 articles, we excluded 5 as they investigated multidomain interventions such as physical exercises or meditation in addition to cognitive training, 2 that used volumetric measures only, 4 using other types of neuroimaging (i.e., not fMRI), and 1 article that grouped patients with Alzheimer's type dementia and those with MCI – since this precluded a clear understanding of the effects in MCI. Thus, a total of 7 studies met the inclusion criteria and they will be discussed according to the questions elaborated for the present review.

## 3. Results

### 3.1. Which Types of COT Have Been Applied to MCI Patients?

Key details for the selected COT studies are presented in Table 1.

#### 3.1.1. Rehearsal-Based Approaches

A total of 3 studies applied rehearsal-based strategies as the primary intervention, all of which used computerized cognitive training. One randomized controlled trial [14] investigated the effects of computerized cognitive training on memory ability in 12 patients with MCI (6 experimental and 6 active control) using a computer-based program developed by Posit Science Corporation (San Francisco, CA). The program included 7 exercises developed to improve processing speed and accuracy in auditory processing, such as (a) determine whether 2 sounds were moving upward or downward, (b) identify a particular syllable while it interrupted a similar sounding syllable, (c) differentiate 2 close sounds, (d) group sounds on a spatial framework, (e) identify 2 similar sounding words, (e) follow instructions with increasing difficulty, and (f) identify the picture that is related to a sentence. The experimental training program was performed for 100 minutes per day, 5 days per week for 24 sessions (2 months on average). The control group underwent 3 computer-based tasks with similar intensity and duration to the experimental program: (a) listening to audiobooks, playing a visuospatial computer game (Myst), and reading newspaper. One differential approach of this study was that the program was performed at the participants' homes on study-provided computers. In addition, participants were contacted weekly to solve problems related to computer-program and other issues. This home approach, on the one hand, allows for better adherence. On the other hand, although the computer-program tasks were highly demanding on speed and accuracy of auditory verbal processing, it did not address memory abilities per se, therefore making it more difficult to recommend it as a memory training program.

Another study of 21 patients with MCI adopted the vision-based speed-of-processing (VSOP) training from the INSIGHT computerized program (Posit Science, San Francisco, CA) with five training tasks: (1) eye for detail, (2) peripheral challenge, (3) visual sweeps, (4) double decision, and (5) target tracker [15]. These tasks focus on speed of processing and attention processes. Patients had to identify what object they saw or where they saw it on the screen. The study included an active control condition involving mental leisure activities (MLA) to control for computer and online experience, such as crossword, Sudoku, and solitaire games. Participants could choose any combination of these games. Both groups were instructed to practice 1 hour per day 4 days per week for 6 weeks in their homes. Although this training program does not focus on memory strategies, the patients benefited from the training in computerized working memory and everyday life activities tasks. Whereas it is a promising approach to offer home-based cognitive training, it requires more sophisticated monitoring procedures.

Recently, 23 MCI patients underwent a computerized cognitive training and a separate group of 14 MCI patients underwent a regime of intense social engagement as a control condition [16]. The computerized cognitive training engaged participants in exercises with multiple cognitive operations including retrieval from memory, management of interference, inhibition, working memory, semantic processing, and logical and abstract reasoning. A total of 20 sessions were completed within a 35-day timeframe (5 sessions a week on weekdays). For the control condition, patients maintained a daily regime of intense social interactions including volunteering work, tour guiding, attending a club, and gardening, according to their personal interests. The control condition had similar duration as the experimental one but was not carried out within a hospital setting as the experimental condition. Although computerized cognitive programs have a number of advantages in clinical research including efficient performance measuring and monitoring of time, as well as type and precision of responses, they are perhaps more susceptible to lower adherence and weaker generalization to everyday tasks.

#### 3.1.2. MST-Based Approaches

Four studies used fMRI to investigate the neurophysiologic changes associated with MST. In the first, Belleville et al. [17] trained 15 MCI patients and 15 healthy controls to use MST during 6 weekly sessions of 120 min each in small groups (4 to 5 participants per group). The main content of the sessions included psychoeducational information regarding memory and ageing, interactive mental imagery, the method of loci, face-name associations, hierarchical organization, and semantic organization techniques. Although this approach has the advantage to offer a short training regimen (6 sessions) and to directly address memory encoding and retrieval processes, it has some limitations in terms of demonstrating which particular strategy contributed to the positive cognitive outcome since a number of different strategies and types of stimuli were used during the 6 sessions.

Hampstead et al. [18] investigated the effects of MST using a face-name association fMRI task in 6 MCI patients. Each participant completed five sessions within two weeks as well as a 1-month behavior-only follow-up [19]. Encoding-related fMRI was acquired pre- and post-training. MST was performed during the three intervening training sessions and required the participants to learn 15 novel face-name associations each session by (1) identifying a salient facial feature, (2) remembering a verbally based “reason” that linked the feature with the name - typically using alliteration, and (3) creating a mental image of the previous two steps. On subsequent trials, participants were required to recall, in order, the feature, the reason, and then the name. For each association, patients were required to spontaneously recall the name on 3 consecutive trials, with a maximum of 10 trials to reach this criterion. An innovative aspect of this study was the implementation of a focused intervention strategy training procedure (face-name association). Within a research investigation context, it has the advantage to reduce confounding factors found in multiple-domain cognitive training with a number of different strategies. Although the number of sessions was reduced (three sessions) due to the study's mechanistic focus, this training protocol can potentially be used in a clinical context together with other strategies to increase the benefits of cognitive training interventions.

In a subsequent single blind randomized controlled study, Hampstead et al. [10] used the same study design and a 3-step MST approach to enhance learning and memory of object location associations (OLAs). A total of 18 patients with MCI and 16 cognitively intact (“healthy”) older adults (HOA) were randomized to either MST or a matched exposure active control group. Participants receiving MST followed the same procedures as above (i.e., feature-reason-image) and were given 9 trials with each of the 45 trained stimuli – the goal of which was to reinforce the use of MST techniques. The exposure control group received the exact same number of training trials and was given corrective feedback after each trial; thus, the only difference between the groups was the addition of mnemonic strategies. In a separate report, the investigators defined the hippocampal region of interest and performed small-volume correction to demonstrate that MCI patients showed the expected pattern of hippocampal hypoactivation at baseline relative to HOA in this OLA paradigm [20].

Balardin et al. [21] investigated the effects of a single session of MST using a word-list paradigm in 18 MCI patients and 17 healthy controls (HC). The MST approach taught participants to organize the words into semantically based categories during encoding and afterwards to retrieve them according to their category. All participants underwent one-day session until they were able to apply the categorization strategy to at least three different word lists. This training approach focused on only one type of strategy which promotes a more effective understanding of the behavior and brain mechanisms related to this training procedure. Since it involves one session, this training strategy can be included in a more extended clinical cognitive training program.

### 3.2. Can Cognitive Training Effects in Patients with MCI Be Generalized/Transferred to Objective Cognitive Measurements?

The majority of the research studies included in this review showed evidence of improved cognitive performance associated with intervention. The intervention effects on neuropsychological tests and cognitive tasks are shown in Table 2. An exception was the De Marco et al. [16] study, in which there were no significant differences between the experimental and control conditions (*p* = 0.136). The authors argued that this lack of significant difference was due to possible insufficient power and reduced exposure to the training regimen (20 sessions in the period of 20 to 35 days, 5 sessions a week, from Monday to Friday); however, this stands in contrast with the above note MST studies that found effects with substantially fewer sessions. Another important factor is the type of outcome measure used in clinical trials of cognitive training (see [9] for a more thorough discussion of this topic). The standardized neuropsychological instruments may not be sufficiently sensitive to capture test-retest changes. Previous studies have shown the importance of including outcome measures with increased ecological validity and consistent with the target of the training, e.g., tasks of face-name association, object location, or semantic organization and processing [17, 18, 20, 21].

Other cognitive outcomes demonstrated the effects of cognitive training involving multiple domains (e.g., speed-processing and attention) on episodic memory tasks including the RBANS (Repeatable Battery for the Assessment of Neuropsychological Status; [14]) and working memory/executive function tasks such as the EXAMINER (Executive Abilities: Measures and Instruments for Neurobehavioral Evaluation and Research; [15]), a computerized test that measures executive function domains including cognitive control (set shifting and flanker tasks), verbal fluency (phonemic and categorical fluency), and working memory (dot counting and 1-back). Such far transfer findings are not well understood, and it has been proposed that they represent possible compensatory effects of the training [14, 15].

Overall, although the majority of the studies in this review found evidence of improvement in objective cognitive measures, there is still a lack of studies showing generalization effects to everyday life activities. Hampstead et al. [18, 20] showed evidence of cognitive improvement after training in tasks similar to everyday life (face-name association and object location) which were related to the training procedure. Lin et al. [15] found improvement after cognitive training on the Instrumental Activities of Daily Living task (IADL), an objective measure of speed and accuracy on multiple instrumental activities of daily living. Future studies should include more ecologically valid outcome measures to identify the benefits of cognitive training in everyday function in persons with MCI.

### 3.3. Do the Observed Changes Represent Evidence of Compensation (i.e., Engaging Alternative Brain Regions or Networks Not “Typically” Engaged) and/or Restoration (i.e., Reengaging the “Typical” Brain Regions or Networks)?

Up to the present time and to the best of our knowledge, only seven randomized or controlled group fMRI studies have investigated the brain regions or networks systems underlying the effects of COTs in persons with MCI. In the last decade, there has been a marked advance in the neuroimaging methods of analyses from comparing the patterns of brain activation through fMRI before and after cognitive intervention to functional brain connectivity. The majority of the studies included in this review showed significant changes in brain activation associated with cognitive training, in typical and atypical brain areas and networks related to memory, suggestive of compensation. Some studies reported functional normalization and possible restoration processes. Changes on brain activation and connectivity related to cognitive intervention reported by these studies are displayed in Table 3.

Rosen et al. [14] and Hampstead et al. [20] reported restoration processes associated with hippocampal activity after cognitive training. The primary cognitive outcome of the latter study revealed that MST improved memory for the trained stimuli significantly more than the matched-exposure condition, regardless of diagnostic status, with benefits persisting at 1 month. Region-of-interest analysis revealed that MCI patients showed the expected pattern of hippocampal hypoactivation at baseline relative to HOA [20] whereas subsequent interaction analyses (i.e., post-training vs. pre-training) revealed that MST partially restored activation in the left hippocampus of MCI patients whereas no changes were evident in the exposure-matched MCI group. Thus, across their two studies, Hampstead and colleagues [18, 20] demonstrated that MST enhanced memory by (re)engaging the lateral frontoparietal cognitive control network as well as the hippocampus.

In Belleville et al. [17], 15 MCI patients and 15 HC underwent learning and training of memory encoding and retrieval strategies. During fMRI scan, participants were instructed to memorize word lists (encoding) and recognize previously studied words amongst a list of new words (retrieval). The authors found increased brain activation after training in typical and atypical memory-related areas, in the frontal, temporal, and parietal areas, particularly in the right inferior parietal lobule, after training, suggesting that their compensatory recruitment was necessary to improve memory performance.

Balardin et al. [21] examined differences in fMRI activation and deactivation patterns during episodic verbal memory encoding in 18 patients with MCI and 17 HC. Participants were scanned before and after one session of cognitive training to apply MST (semantic clustering) during encoding of word lists. After training, greater recruitment of frontoparietal regions, especially in the left hemisphere, was observed in both MCI and HC associated with improvement in memory performance. Moreover, controls showed negative-going BOLD (i.e., reduced activation) of the medial prefrontal cortex and right superior frontal gyrus during encoding after training. MCI patients demonstrated a pattern of less deactivation in these regions which are related to the DMN. These findings provide evidence of differences in brain activation and deactivation patterns and brain compensation mechanisms after cognitive training in MCI and HC persons probably related to the encoding deficits commonly found in MCI.

Lin et al. [15] investigated changes in brain functional connectivity after cognitive training in 21 MCI patients. The experimental group underwent Vision-Based Speed-of-Processing Training (*n* = 10; INSIGHT online program from Posit Science, San Francisco, CA), and the control group Mental Leisure Activities Control (*n* = 11; online crossword, Sudoku, and solitaire games). The experimental group showed reduced central executive network connectivity possibly related to reduced frontal lobe–oriented dependence and better efficiency of information processing. They also reported maintenance of DMN connectivity, which was viewed as a positive outcome since progressive decrease was expected in MCI.

De Marco et al. [16] included 23 MCI patients allocated to the experimental condition of one-month computerized exercises (memory retrieval, inhibition, working memory, and logical reasoning) and 14 MCI patients to the control condition (intense social engagement). They found increased upregulation of connectivity of the DMN in left parietal regions after cognitive training that was interpreted as compensatory in nature and occurred despite a lack of improvement in cognitive functioning.

## 4. Discussion

Overall, the results of studies evaluating the efficacy of COTs indicate that persons with MCI benefit from COTs, with evidence of direct training gains and some transfer effects. In addition, MCI individuals are able to systematically practice cognitive tasks and learn several strategies to optimize cognitive functioning. The neuroimaging findings showed that COTs frequently led to an increase in brain activation (particularly in frontoparietal regions) and either an increase or maintenance in connectivity. The available evidence suggests that the brain remains highly plastic in those with MCI and that neuroimaging is sensitive to change after COTs, thereby suggesting that neuroimaging can reasonably serve as an outcome measure for interventional studies.

Based on our previous methodological review [9], we categorized the COTs analyzed here in rehearsal- and MST-based approaches. The rehearsal-based approach relies on the repetition of information over time, the MST-based approach on learning new skills or strategies to compensate and/or optimize cognitive functioning. In our review, both approaches led to significant cognitive improvements at post-training, although the rehearsal-based approach showed more conflicting evidence, since there were negative findings [16] and limitations regarding transfer effects [14]. However, there was some indication of transfer effects in at least one study [15] and most reported significant neuroimaging changes after training. Contrary to our expectations, we did not find a repetition suppression effect (i.e., decrease of activation), since one study reported increased hippocampal activity after auditory-verbal computerized training and others reported increase or maintenance of connectivity after multicognitive computerized training [15, 16]. It is worth mentioning that these studies applied an intense regimen of training, such as 20-24 sessions, 4-5 times per week. Given the lack of information about dose-response relationships (see [9]), it is possible that such extended training paradigms may have different effects than those in which the exact same stimuli are repeated (i.e., stimulus specific effects). Future studies should investigate such possibilities more systematically, especially as they relate to task- and resting-state-related changes, since the nature of contrast- and connectivity-based fMRI is inherently different.

All the studies related to the MST-based approach reported significant cognitive improvement and increased activation in lateral frontoparietal regions. This finding is in line with the fact that MST requires the user to actively hold and manipulate to-be-learned information, a process that engages cognitive control mechanisms such as working memory. Moreover, critical areas relevant to memory processing (e.g., hippocampus) also showed increased activation after training [20]. Together, these findings suggest that MST enhances functioning in memory-related networks.

The current literature suggests that changes after COTs can represent both restoration and compensation. This conclusion is consistent with the Interactive model proposed by Belleville and colleagues [22] that suggests training-induced activation changes depend on a number of interacting factors, including the format and characteristics of the training. Theoretically, any intervention that clearly engages a particular cognitive process (e.g., cognitive control) should induce change in brain region(s)/network(s) that mediate that process (e.g., lateral frontoparietal cortex). The nature of this change may well depend on the baseline pattern of activity/connectivity. Restoration of functioning would be suggested by hypoactivity/connectivity at baseline with increased activity/connectivity after intervention. Our findings of hippocampal change [20] are a good example of this since patients showed less activation than cognitively intact controls at baseline AND then an increase in activation after MST. In contrast, compensation would be supported by patients showing intervention-induced change in areas not engaged by cognitively intact older adults after comparable intervention. Belleville and colleagues' [17] findings of increased right parietal activation after MST provide a good example of this since patients did not show regional hypoactivation of this area at baseline and cognitively intact participants did not engage this area after MST. Intervention-related reduction in activation/connectivity would presumably occur if the trained task/stimuli were exactly the same as that used in the scanner (e.g., classic repetition suppression effects) or truly enhanced efficiency (e.g., cognitive improvement within the context of reduced activation/connectivity).

Limitations of this review include the following. First, all of the studies classified the MCI individuals based on clinical/cognitive criteria but none included biomarker data (e.g., beta amyloid or tau levels). While beneficial for general clinical practice, it is difficult to know whether these groups represent a uniform etiology (e.g., Alzheimer's disease). This aspect is particularly relevant given the longitudinal nature of intervention trials and neuroimaging-related changes that are attributable to the development and worsening of Alzheimer's disease versus other processes (e.g., vascular disease). Second, although the majority of the studies in this review found evidence of improvement in objective cognitive measures, there is still a lack of measures specifically designed to evaluate the cognitive process trained, and, critically, transfer effects to everyday life activities. Future research should develop and validate new tools that better emulate real-world problems that patients experience. We believe that technologies such as virtual reality hold promise in this regard. Third, most of the studies included are based on small sample sizes, since the range of MCI participants in the experimental group was 6 to 17 individuals. Although the current evidence is encouraging, the fact that there are only 7 studies limits definitive conclusions.

In conclusion, this review provides some initial understanding of the impact of COTs on cognition and brain mechanisms in individuals with MCI. The efficacy of the COTs will be enhanced if future studies replicate the current methodologies in larger samples and/or apply the same programs in different samples and sites.

## Figures and Tables

**Table 1:** Types of COT applied to MCI patients.

Study	Design study	Sample	COT	Cognitive domains trained	CCI	Intervention format	Outcome measures	fMRI protocol	Intervention fMRI results on MCI	MCI COT clinical results
Rosen et al. [23]	RCT double-blind	12 MCIs 6 COT MCI Age/education: 70.7/16.7 6 CCI MCI Age/education: 70.0/13.4 Petersen criteria	Computer-based auditory processing training by Posit Science Corporation	Auditory, accuracy, processing speed, immediate and working memory	Audio books, online newspaper Myst game.	Home-based computer-assisted for COT & CCI 24 sessions 5 times/week 90–100 min/session	Task-related hippocampal functional activity changes; performance at RBANs, and relationship with brain activity	Auditory verbal task with discrimination of concrete from abstract words	Increased activation on L hippocampus after COT and decreased activation in this area after CCI. L hippocampal activation was associated with immediate memory on RBANs.	COT MCI showed better gain in immediate memory performance on RBANs compared to CCI MCI.

Belleville et al. [17]	CGT Single–blind	15 COT HC Age/education: 70.0/13.4 15 COT MCI Age/education: 70.1/13.7 Petersen criteria	Visual imagery Method of loci Face-name association Hierarchical and semantic organization	Episodic memory and executive functions	No	Group sessions 6 sessions 1 times/week 120 min/session	Task-related whole-brain functional activity changes; performance at fMRI task, and off-scan word list recall	Verbal memory encoding and retrieval of a word list	MCI COT increased activation in R inferior parietal lobule, frontal gyrus, R cerebellum, and basal ganglia on encoding and increased activation in R/L superior temporal gyrus, L frontal, and parietal cortex on retrieval. Activation in R parietal lobule was associated with better performance on delayed word recall.	Improvement in performance on the retrieval scan task and on word list recall. Better performance on immediate recall compared with delayed recall

Hampstead et al. [18]	Case series	6 COT MCI Age/education: 73.5/15.7 Petersen criteria	Explicit memory strategy: visually identify a facial feature and then link it to a phonological cue to recall the associated name	Associative episodic memory	No	Individual sessions 5 sessions 2 weeks	Task-related whole-brain functional activity and connectivity; performance on face-name recognition off-scan task	Associative encoding of face name pairs	COT-specific activations in L temporoparietal junction, L frontal operculum, L temporal cortex, and R/L medial areas of frontal, parietal, and occipital cortices. Increased connectivity in lateral frontal and parietal regions	Improvement on memory performance for TS and US stimulus after COT. Better performance and fast reaction times in recognition of TS

Hampstead et al. [20]	RCT single blind	16 HC vs. 18 MCI 8 COT HC Age/education: 72.1/15.8 8 CCI HC Age/education: 72.1/16.5 9 COT MCI Age/education: 71.7/17.4 9 CCI MCI Age/education: 70.8/16.8 Petersen criteria	Explicit memory strategy: identify a salient feature within the room near the object, use a verbally based “reason” that related the object to the specific feature, and then form a corresponding mental image	Associative episodic memory	Repeated exposition of stimuli	Individual sessions for COT & CCI 5 sessions 2 weeks	Task-related hippocampal functional activity; performance on-scan retrieval task	Associative encoding and retrieval of object location association task	COT aMCI showed increased activation in L hippocampal body on encoding of TS and US. Increased activation in R/L hippocampal body and tail on retrieval of TS and increased activation in R hippocampus for US	COT groups showed better performance on-scan retrieval task for TS compared to CCI groups.

Balardin et al. [24]	CGT single blind	18 COT HC Age/education: 68.25/11.19 17 COT MCI Age/education: 69.5/9.2 17 COT MCI Age/education: 69.5/9.2 Petersen criteria	Semantic strategic training: organization of word lists into categories during encoding and retrieval according to their category	Episodic memory and executive functions	No	Individual session 1 session 30 min/session	Task-related whole-brain functional activity; performance at SR and UR word lists, recall, and relationship with brain activity	Verbal memory encoding of SR and UR word lists	COT MCI showed increased activation in frontoparietal network regions including L DLPFC and L VLPFC and decreased activations on the occipital cortex. Increased activation on R superior frontal gyrus and vmPFC cortex related to semantic strategy implementation. Better performance on strategic clustering was predictive of increased activation on OFC cortex.	Improvement on performance on word list recall and on semantic strategy implementation after COT. Better recall on the SR list compared to UR list.

Lin et al. [15]	RCT single blind	21 MCI 10 COT MCI Age/education: 1 72.9/1 11 CCI MCI Age/education: 73.1/5 Petersen criteria	Vision-based speed of processing training online program by Posit Science Corporation	Visual accuracy, processing speed attention, and working memory	Computer-based activities: online crosswords, sudoku, and solitaire games	24 sessions 6 weeks 4 times/week 60 min/session Home-based computer-assisted	Connectivity in DMN and CEN; performance at visual processing speed, working memory, verbal fluency, and IADL tasks	Resting state	Increased connectivity on CEN after COT. COT MCI showed increased connectivity on DMN compared to CCI MCI.	Improvement on performance on visual processing speed and attention test, and also on transfer domains tests related to working memory and IADLs after training

De Marco et al. [16]	RCT single blind	37 MCI 23 COT MCI Age/education: 73.74/8.7 14 CCI MCI Age/education: 73.74/10.5 Petersen criteria	Cognitive training package	Semantic processing, reasoning, executive functions, and episodic memory retrieval	Social interaction activities: volunteering, tour guiding, attending a club, or gardening	Home-based computer-assisted 20 sessions 5 times/week 60–90 min/session	Connectivity in DMN - performance at CCRI and relationship with brain activity	Resting State	Increased connectivity on Precuneus – Cuneus after COT. Enhances on CCRI were associated with increased connectivity on parietal DMN	No improvement verified on neuropsychological measures applied

RCT: randomized controlled trial; CGT: controlled group trial; COT: cognitive oriented treatment; CCI: control condition intervention; MCI: mild cognitive impairment; HC: healthy controls; fMRI: functional magnetic resonance imaging; TS: trained stimulus; US: untrained stimulus; SR: semantic related word list; UR: unrelated word list; R: right; L: left; DLPFC: dorsolateral prefrontal cortex; VLPFC: ventrolateral prefrontal cortex; vmPFC: ventromedial prefrontal cortex; OFC: orbitofrontal cortex; CEN: central executive network; DMN: default mode network; IADLs: instrumental activities of daily living; CCRI: composite cognitive change ratio index.

**Table 2:** COT effects on neuropsychological tests.

Study	Design	Intervention conditions	Sample	Cognitive measures	Cognitive task results after training
Rosen et al. [14]	RCT double-blind	Auditory processing training Computer-based activities	COT MCI CCI MCI	RBANS scores	NS
				RBANS immediate memory COT MCI > CCI MCI	*p* = 0.027; Cohen's *d* = 1.38

Belleville et al. [17]	CGT single–blind	Mnemonic strategy training	COT HC COT MCI	Word list recall MCI = HC	*p* < 0.05, *η*^2^ = 0.21
				MCI < HC	*p* < 0.05, *η*^2^ = 0.16
				Word list Immediate recall MCI = HC	*p* < 0.001; *η*^2^ = 0.73
				Performance on FMRI scan MCI = HC	*p* < 0.01, *η*^2^ = 0.23

Hampstead et al. [18]	Case control	Face name Association strategy	COT MCI TS US	Post fMRI scan recognition task TS = US	*p* = 0.001
				TS > US	*p* = 0.002
				Reaction time TS	*p* = 0.04

Hampstead et al. [20]	RCT single blind	Object location Association training Stimuli Exposition	COT HC COT MCI CCI HC CCI MCI	Object location Recognition task TS-COT group > CCI group	*p* = 0.026, *pη*^2^ = 0.155
				TS-HC > MCI	*p* < 0.001, *pη*^2^ = 0.343
				US-HC > MCI	*p* < 0.001, *pη*^2^ = 0.314

Balardin et al. [21]	CGT single blind	Semantic encoding strategy training	COT HC CCI MCI	Word list free Recall HC > MCI	*p* = 0.001
				MCI = HC	*p* < 0.001
				SR > UR	*p* < 0.001
				Semantic cluster HC = MCI	*p* = 0.272
					*p* < 0.001
				Mean number of clusters MCI < HC	*p* = 0.047

Lin et al. [15]	RCT single blind	Visual speed of processing and attention training Computer-based activities	COT MCI CCI MCI	UFV-reaction time COT MCI > CCI MCI	*p* = 0.02, *η*^2^ = 0.26
				Working memory COT MCI > CCI MCI	*p* = 0.01, *η*^2^ = 0.28
				Cognitive control COT MCI > CCI MCI	*p* = 0.03, *η*^2^ = 0.21
				Verbal fluency	NS
				IADL completion time	NS

De Marco et al. [16]	RCT single blind	Cognitive training Social engagement	COT MCI CCI MCI	CCRI	NS

RCT: randomized controlled trial; CGT: controlled group trial; COT: cognitive oriented treatment; CCI: control condition intervention; MCI: mild cognitive impairment; HC: healthy controls; TS: trained stimulus; US: untrained stimulus; SR: semantic related; UR: unrelated; UVF: used field of view; IADLs: instrumental activities of daily living; CCRI: cognitive change ratio index; *p*: *p* values; *η*^2^: eta-squared; *η*^2^: partial eta-squared; NS: not significant results.

**Table 3:** COT BOLD activations and connectivity.

Study	Design	Sample	Intervention conditions	fMRI protocol cognitive measure	Post-intervention fMRI comparisons	Direction of FMRI	fMRI results after intervention	fMRI and cognitive tasks
Rosen et al. [14]	RCT double-blind	12 MCIs	Auditory processing training Computer-based activities	Auditory verbal task RBANS	COT MCI	Increasing	L hippocampus	L hippocampus activation correlated with changes in memory performance on RBANS (*r* = 0.49)
					CCI MCI	Decreased	L hippocampus	

Belleville et al. [17]	CGT single-blind	15 HC 15 MCI	Mnemonic strategy training	Word list memory encoding and retrieval and word list recall test	ENCODING HC	Decreased	R/L basal ganglia, R/L cingulate gyrus, R inferior frontal gyrus, R inferior and superior parietal cortex, R inferior, medial and superior frontal gyrus, L prefrontal cortex, L precentral gyrus, and R hippocampus	HC performance on immediate word recall was correlated with activation in L inferior frontal gyrus during retrieval (*r* = 0.521)
					MCI	Increased	L superior temporal gyrus, L thalamus, putamen and globus pallidus, R inferior parietal cortex, R superior frontal gyrus, and R cerebellum	
					RETRIEVAL HC	Increased	R middle temporal gyrus, thalamus, R superior temporal gyrus, R putamen, R/L precuneus, L superior temporal gyrus, L inferior frontal gyrus, and R hippocampus	
					MCI	Increased	L postcentral gyrus, L inferior parietal lobule, L inferior and supramarginal gyrus, R/L posterior cingulate, R/L superior temporal gyrus, R insula, and L middle frontal gyrus	MCI performance on delayed word recall was correlated with activation in R inferior parietal lobule during encoding (*r* = 0.538)
					COT MCI post > pre encoding		Cingulate and medial frontal gyri (*p* < 0.001) and R inferior parietal lobule (*p* < 0.01)	
					Retrieval		L middle frontal gyrus (*p* < 0.01) R superior Parietal lobule (*p* < 0.05)	

Hampstead et al. [18]	Case control	6 MCI	Face name associative training	Face name Associative encoding and off-scan recognition task	TS > US	Increased	R/L medial frontal cortex; medial parietal cortex, precuneus, medial occipital cortex, L frontal operculum, L temporoparietal junction, and L temporal cortex	
					U S > TS	Increased	L middle occipital gyrus	
					U S > RS	Increased	L occipital cortex, L inferior frontal cortex and R/L inferior parietal cortex. The activations on inferior frontal gyrus, inferior frontal sulcus, superior middle occipital gyrus and fusiform area could be related to attempts to generalize the trained strategies	
					Connectivity analysis		Effective connectivity on L middle temporal gyrus	

Hampstead et al. [20]	RCT single-blind	16 HC 18 MCI	Object location Associative training Stimuli exposition	Object location associative encoding and retrieval task	ENCODING COT MCI	Increased	L hippocampal body (TS + US)	
					RETRIEVAL COT HC	Increased	L hippocampal tail and R hippocampal head (US)	
					COT MCI	Increased	R/L hippocampal body and tail (TS)	
						Increased	L hippocampal body and tail (US)	
					COT HC + CCI MCI	Decreased	R hippocampal body (TS)	
					COT MCI > CCI MCI	Increased	L hippocampal body and R hippocampus (TS)	
						Increased	R hippocampal body (US)	

Balardin et al. [21]	CGT Single Blind	17 MCI 18 HC	Semantic encoding strategy orientation	SR and UR Word Lists memory encoding with off-scan free recall	HC	Increased	L middle frontal gyrus, inferior frontal gyrus, dorsal premotor cortex, posterior parietal cortex, angular gyrus within intraparietal sulcus borders	Performance on encoding the SR list was correlated with activations in orbitofrontal cortex, medial prefrontal cortex, and anterior cingulate HC - higher performance correlated with greater decrease on activations (*r* = −0.734) MCI - higher performance correlated with greater increase on activations (*r* = 0.339)
						Decreased	R superior frontal gyrus, vmPFC, L inferior Parietal Cortex, infero-lateral Temporal cortex, posterior cingulate and precuneus	
					MCI	Increased	L middle frontal gyrus, inferior frontal gyrus, dorsal premotor cortex, posterior parietal cortex, angular gyrus within intraparietal sulcus borders	
						Decreased	Parietooccipital cortex	
					Changes on activations HC	Decreased	L middle frontal gyrus	
						Decreased	R superior frontal gyrus	
					MCI	Increased	R superior frontal gyrus	

Lin et al. [15]	RCT	21 MCI	Visual	Resting	Connectivity		Improvement	
	Single-blind		Speed of processing and attention training Computer-based activities	State	Analysis COT MCI		of connectivity on CEN (*p* = 0.02)	
					CCI MCI		Trend to poor connectivity strength in DMN (*p* = 0.07)	
					COT MCI > CCI MCI		COT MCI showed increased connectivity in DMN compared to CCI MCI (*p* = 0.004, *η*^2^ = 0.62)	

De Marco et al. [16]	RCT single-blind	23 MCI	Cognitive training Social engagement	Resting state and CCRI	DMN connectivity analysis COT MCI		Increased in DMN connectivity on precuneus - cuneus and increased connectivity on R/L parietal cortices	Connectivity on parietal DMN were associated with the CCRI (*r* = 0.409)
					CCI MCI		Decreased connectivity in R/L parietal cortices	

RCT: randomized controlled trial; CGT: controlled group trial; COT: cognitive oriented treatment; CCI: control condition intervention; MCI: mild cognitive impairment; HC: healthy controls; fMRI: functional magnetic resonance imaging; TS: trained stimulus; US: untrained stimulus; SR: semantic related; UR: unrelated; R: right; L: left; vmPFC: ventromedial prefrontal cortex; r: Pearson correlation coefficient; CCRI: cognitive change ratio index; DMN: default mode network.
